# *Pediococcus acidilactici* Long21 Alleviates Intestinal Sensitivity and Improves Gut Health in German Shepherd Dogs by Regulating Intestinal Metabolic Profiles

**DOI:** 10.3390/ani16203181

**Published:** 2026-10-10

**Authors:** Xinyu Zhang, Yuhe Ma, Haozhen Liu, Ying Yang, Zhenlong Wu

**Affiliations:** State Key Laboratory of Animal Nutrition and Feeding, College of Animal Science and Technology, China Agricultural University, Beijing 100193, China; zhangxinyu951105@163.com (X.Z.);

**Keywords:** dog, intestinal sensitivity, *Pediococcus acidilactici*, nutrient digestibility

## Abstract

**Background:** Canine intestinal sensitivity is a multifactorial functional gastrointestinal disorder characterized by recurrent soft feces, impaired nutrient utilization, and low-grade intestinal inflammation, and it acts as a major trigger for chronic gastrointestinal discomfort in dogs. Conventional antibiotic therapy causes side effects and drug resistance. Probiotics modulate host physiology, immunity, and metabolism, serving as promising alternatives for improving canine intestinal health. This study aimed to compare the gut microbiota of healthy and sensitive German shepherd dogs, screen and identify candidate probiotics, and verify their efficacy in ameliorating canine intestinal dysfunction and hypersensitivity. **Results:** Fecal microbiota transplantation from healthy dogs effectively improved these fecal indicators and was associated with enhanced serum antioxidant and anti-inflammatory capacities in intestinal-sensitive dogs (*p* < 0.05). Additionally, 16S rRNA sequencing revealed that healthy German Shepherd dogs had higher intestinal microbial α- and β-diversity, with *Pediococcus acidilactici* enriched in healthy dogs and *Shigella* increased in diarrheic dogs (*p* < 0.05). *P. acidilactici* Long21 intervention, either alone or combined with its fermentation supernatant, significantly improved fecal traits; reduced pathogenic bacterial abundance; increased the apparent digestibility of dry matter, crude fibre, and ether extract; and was associated with normalized antioxidant function and inflammatory indicators in German Shepherd dogs with intestinal sensitivity (*p* < 0.05). We identified 2085 differentially abundant metabolites enriched in amino-acid and energy metabolism using untargeted metabolomics, with glutamate, malate, and pyruvate as hub metabolites correlated with nutrient digestibility and fecal traits. Conclusions: Intestinal flora imbalance is associated with intestinal sensitivity-related diarrhea in dogs. *P. acidilactici* Long21 supplementation alleviated intestinal sensitivity-associated diarrhea and improved intestinal health, possibly through coordinated changes in gut microbiota, metabolic profiles, and nutrient utilization. These findings provide a theoretical basis and a candidate strain for microecological intervention, although causal mechanisms remain to be confirmed.

## Simple Summary

Some pet dogs experience recurrent loose stools, poor nutrient absorption, and mild gut inflammation, collectively known as intestinal sensitivity. The use of antibiotics to treat this issue carries a risk of side effects and antibiotic resistance. This study was conducted to compare gut microbes between healthy and sensitive German Shepherd dogs and test a candidate probiotic. We found that sensitive dogs had less diverse gut microbes. A probiotic called *Pediococcus acidilactici* Long21 improved stool quality, reduced harmful bacteria, and boosted nutrient digestion, with linked changes in gut metabolites. This probiotic may help ease canine intestinal discomfort, offering a natural gut-support option for pet dogs, though the exact causal mechanisms remain unproven.

## 1. Introduction

Dogs are one of the earliest animals domesticated by humans; they have provided companionship and been indispensable to work and public safety for thousands of years. With the development of society and improvements to living standards, the number of companion dogs has continued to increase globally, and their health and welfare have received increasing attention [1,2]. Gastrointestinal diseases, characterized by diarrhea, vomiting, and decreased appetite, are particularly common, seriously affecting the health and quality of life of companion animals. Chronic gastrointestinal diseases, such as canine sensitive intestinal disease and inflammatory bowel disease (IBD), are important causes of persistent or recurrent diarrhea, and their occurrence is closely related to chronic inflammation of the intestinal mucosa, immune disorders, and intestinal barrier damage [3,4,5]. Canine intestinal sensitivity represents a functional gastrointestinal disorder with multifactorial etiology rather than a single symptom of simple diarrhea. Its clinical manifestations include intermittent soft-formed or loose stools, increased fecal moisture, variable fecal scores, occasional flatulence, decreased nutrient digestibility, and low-grade intestinal inflammation in the absence of confirmed primary viral, bacterial, or parasitic enteropathogens. Recurrent loose stools or diarrhea are the most prominent clinical sign, but affected dogs may also show subtle subclinical changes without overt watery diarrhea.

Studies have shown that the imbalance in the intestinal flora structure is a key factor driving the occurrence and development of chronic intestinal inflammation and diarrhea in dogs [6,7]. Diarrheic dogs often show decreased diversity of intestinal microorganisms, reduced abundance of beneficial bacteria, and increased abundance of pathogenic bacteria, which further aggravates intestinal inflammatory responses, damages the mucosal barrier function, and forms a vicious cycle of inflammation and flora disorder, ultimately leading to persistent diarrhea [8,9]. Traditional treatments, like immunosuppressive glucocorticoids and antibiotics, are effective in relieving symptoms to a certain extent. However, their long-term use is prone to cause immunosuppression, secondary infections, and bacterial resistance, limiting their application in clinical practice [10,11]. Therefore, searching for safe, effective, and long-term intervention methods is of great clinical significance for dogs affected by intestinal sensitivity and diarrhea.

Probiotics, as a type of live microbial preparation that can regulate the intestinal microecology, have been widely confirmed to maintain intestinal homeostasis by inhibiting the colonization of pathogenic bacteria, enhancing intestinal barrier function, regulating local immune responses, and improving the metabolic product spectrum [12,13,14]. They have become a research focus due to their distinctive advantage of employing a “bacteria-against-bacteria” approach. Probiotics can effectively improve the intestinal flora structure, alleviate intestinal inflammation, relieve clinical symptoms such as diarrhea and loose stools, and show good application potential in the management of sensitive intestinal and chronic enteritis-related diarrhea [15,16,17]. Among these strains, *P. acidilactici* stands out as a promising candidate, owing to its distinctive characteristics, including resistance to gastric acid and bile salts, the ability to produce pediocin, as well as rapid oxygen consumption and pH reduction [18,19]. In vitro studies have shown that probiotics can inhibit intestinal pathogens such as *Escherichia coli*, *Salmonella* and *Staphylococcus aureus* in multiple ways by competing for adhesion sites and secreting bacteriocins and organic acids [20]. *P. acidilactici* can also promote immunoglobulin A (IgA) and β-defensin secretion, thereby enhancing mucosal immunity in farm animals [21].

Despite these advantages in intestinal health, the application of *P. acidilactici* in dogs has not yet been studied. Therefore, this research focuses on the issue of diarrhea caused by the sensitive intestines of dogs and explores the beneficial effect of *P. acidilactici* Long21 on intestinal microecology, intestinal function, and antioxidant capability, with the aim of providing the scientific basis for the safe prevention and maintenance of intestinal health in dogs with sensitive intestines.

## 2. Materials and Methods

### 2.1. Fecal Sample Collection

Fecal samples were collected from June 2023 to December 2025 at the Veterinary Teaching Hospital in Beijing. A total of 18 German shepherd dogs with intestinal sensitivity and 7 clinically healthy German shepherd dogs were enrolled in this study. All enrolled dogs had completed vaccination with the Pfizer Purevax Canine Pentavalent Vaccine. Subjects were client-owned German Shepherd dogs with spontaneous non-infectious intestinal sensitivity. Dogs diagnosed with intestinal sensitivity in this study fulfilled the following composite clinical criteria: persistent or recurrent loose stools with Waltham faecal score ≥ 3 for at least 14 days; elevated faecal moisture content; reduced apparent nutrient digestibility; no history of recent antibiotic or steroid administration within one month before enrolment. Sample recruitment was constrained by strict inclusion–exclusion criteria and owner availability. A priori power analysis was not performed. Post-hoc effect sizes (Cohen’s d) were calculated for major endpoints; most significant variables showed moderate-to-large effect sizes (Cohen’s d > 0.5). All experimental conditions including diet, housing, scoring criteria and assay kits were standardized to minimize extrinsic variation among individual dogs.

All dogs were fed a commercial chicken-brown-rice complete dog diet (Fupaise Co., Ltd., Shanghai, China). Full ingredient information and proximate nutritional composition are provided in Supplementary Table S1. Each dog was individually housed. The weight of offered diet was recorded daily. Uneaten residual feed was collected and weighed each morning prior to feeding. Daily feed consumption was calculated by subtracting leftover feed weight from the weight of provided feed. Feed intake data were continuously recorded during both the acclimation and formal experimental periods for subsequent apparent nutrient digestibility calculation. Written informed consent was obtained from all pet owners prior to sample collection. Fresh fecal samples were screened for common diarrhea-associated viruses and parasites; only samples yielding negative pathogen results were used for downstream processing. Real-time qPCR detection of canine parvovirus (CPV) and canine coronavirus (CCoV) was carried out with the QubeMDx+™ Canine GI Assay (Cat.No. PCRCQ3112, Credo Diagnostics, Murphy, TX, USA), following the manufacturer’s protocols, with each sample assayed in technical duplicates. Parasite screening was performed using saturated sodium-chloride flotation combined with light-microscopic examination. Subsequently, fresh feces were homogenized and filtered to remove large particulate debris. Sterile glycerol was added to the filtered fecal suspension to achieve a final concentration of 30% (*v*/*v*). The resulting suspension was aliquoted and stored at −80 °C until preparation for fecal microbiota transplantation (FMT).

### 2.2. Experimental Animals and Design

All experimental animal protocols in this study were approved by the Animal Care and Use Committee of China Agricultural University (AW02504202-1-10). After a 6-day acclimation period, 25 German shepherd dogs were divided into a normal fecal morphology group (Normal, n = 7) or abnormal fecal morphology (Diarrhea, n = 18), based on fecal morphology. Pathogen screening of faecal samples was performed before the experiment. The main target enteric pathogenic bacteria were *Escherichia coli* and *Shigella* spp. Fresh faecal suspensions were serially diluted and spread onto MacConkey agar plates. After incubation at 37 °C for 24 h under aerobic conditions, typical lactose-fermenting colonies were counted to determine the abundance of *Escherichia-Shigella*. All German shepherd dogs were housed in the same environment and fed a commercial diet with the same batch number for 7 days. Fecal samples were collected for moisture content, the number of pathogenic bacteria in the fecal, and the digestibility of nutrients analysis.

### 2.3. Fecal Microbiota Transplantation (FMT) and Sample Collection

To further verify whether intestinal microbiota serves as a key determinant of intestinal physiological function, fecal microbiota transplantation was performed. Donor screening criteria: Healthy German Shepherd dog donors (n = 7) were clinically assessed; donors exhibited normal faecal consistency (Waltham faecal score = 2–2.5), no history of gastrointestinal disease, no antibiotic or probiotic exposure within 3 months prior to sample collection, negative qPCR results for canine parvovirus and canine coronavirus, and negative findings for intestinal parasites by saturated sodium-chloride flotation microscopy.

Fresh faecal material collected from eligible donors was homogenized, filtered to remove large particulate debris, mixed with sterile glycerol to a final concentration of 30% (*v*/*v*), aliquoted, and stored at −80 °C. Frozen faecal inoculum (not fresh material) was used for transplantation in this study. Each recipient dog received faecal suspension derived from 5 g of original donor faeces, resuspended in sterile physiological saline to a final volume of 20 mL. The FMT suspension was administered via oral gavage. Administration frequency: once daily for consecutive 21 days. Sixteen diarrheic German Shepherd dogs were randomly divided into two groups: one group was orally given an equal volume of sterile saline (Con group), and the other group received the faecal microbiota suspension (FMT group) following the above-mentioned protocol. Fecal and serum samples were collected at pre-FMT, day 14 and day 21 of intervention for downstream analyses [22].

### 2.4. Isolation and Beneficial Effects of P. acidilactici Long21 in Dogs

A strain of *P. acidilactici* Long21 was isolated from the intestinal tract of healthy German shepherd dogs using de Man, Rogosa and Sharpe (MRS) medium and subsequently employed to verify the proposed research hypothesis. Eighteen German Shepherd dogs with intestinal sensitivity were randomly divided into three groups. Dogs in the Con group were fed 50 mL sterile physiological saline; animals in the *P. acid* group were fed 50 mL liquid containing fermentation supernatant plus viable *P. acidilactici* Long21 (daily dose: 2.1 × 10^9^ CFU viable bacteria); animals in the C. P group were fed 50 mL liquid with fermentation supernatant removed but containing an equal amount of viable bacteria (2.1 × 10^9^ CFU per dog per day). The viable count of *P. acidilactici* Long21 was determined by plate counting on MRS agar before administration. All probiotic suspensions and the sterile saline control were administered by oral gavage once daily to ensure accurate delivery of the exact daily dose to each individual dog.

### 2.5. Determination of Biochemical Indicators in Serum

Whole blood was collected from the forelimb vein using serum-separation vacuum tubes. Blood samples were allowed to clot at room temperature for 30 min, followed by centrifugation at 3000× *g* for 15 min at 4 °C. The obtained serum was aliquoted and stored at −80 °C for subsequent biochemical analysis. Serum concentrations of hydrogen peroxide, catalase, malondialdehyde, glutathione S-transferase and myeloperoxidase were quantified using commercially available assay kits from Nanjing Jiancheng Bioengineering Institute (Nanjing, China), following the protocols provided by the kit manufacturer.

### 2.6. Apparent Digestibility

The fecal output of each group of dogs was weighed at 8:00 daily. The collected fecal samples were placed in an oven at 65 °C for overnight drying and subsequently crushed and mixed well to prepare air-dried samples following a specific air-drying process. The treated samples were kept in a cool and dry place.

At the end of the sampling period, the nutrient content of the feed and manure air-dried samples was determined. This included ashing the samples to constant weight using the GB 50094-2010 [23] scorch weight method at a high temperature of 500–600 °C to obtain crude ash; extracting crude protein from crude ash using the GB 50095-2016 [24] Kjeldahl nitrogen determination method; obtaining crude fat using the petroleum ether reflux leaching method; and quantifying the crude fat by the GB 50096-2011 [25] extraction method.

Finally, the apparent digestibility of nutrients was calculated. This is obtained by subtracting the mass of a nutrient excreted through feces from the total amount of a nutrient in the feed ingested by the animal, dividing by the total amount of that nutrient ingested, and multiplying by 100%.

### 2.7. Untargeted Fecal Metabolomics

Untargeted metabolomics analysis was performed on dog faecal samples. Briefly, metabolites were extracted using a pre-cooled methanol–acetonitrile–water solvent with an internal standard. The extracts were analysed by UHPLC-Q Exactive Orbitrap MS in both positive and negative ESI modes. QC samples were injected regularly to monitor instrument stability. Raw data were processed for peak alignment and normalization. Metabolite annotation was performed following MSI confidence levels. A total of 2085 differential metabolites were obtained. Wilcoxon rank-sum test was used for group comparison. The Benjamini–Hochberg method was applied for FDR correction of multiple testing. Metabolites with FDR < 0.05 were considered significantly different. PCA and PLS-DA were used for multivariate analysis, and KEGG pathway enrichment was conducted.

### 2.8. Statistical Analysis

Raw data were compiled in Microsoft Excel. Statistical analyses were performed using SPSS 23.0. All results are presented as mean ± standard error of the mean (SEM). One-way analysis of variance was applied to detect intergroup differences, followed by Duncan’s post-hoc multiple comparison test. A *p*-value below 0.05 was defined as the threshold for statistical significance. Figures were generated with GraphPad Prism 9.0 (Graph Pad Software Inc., San Diego, CA, USA).

## 3. Results

### 3.1. Comparative Study on Fecal Scores, the Number of Pathogenic Bacteria in the Feces, and the Digestibility of Nutrients Between Healthy Dogs and Those Affected by Intestinal Sensitivity

**Figure 1:**
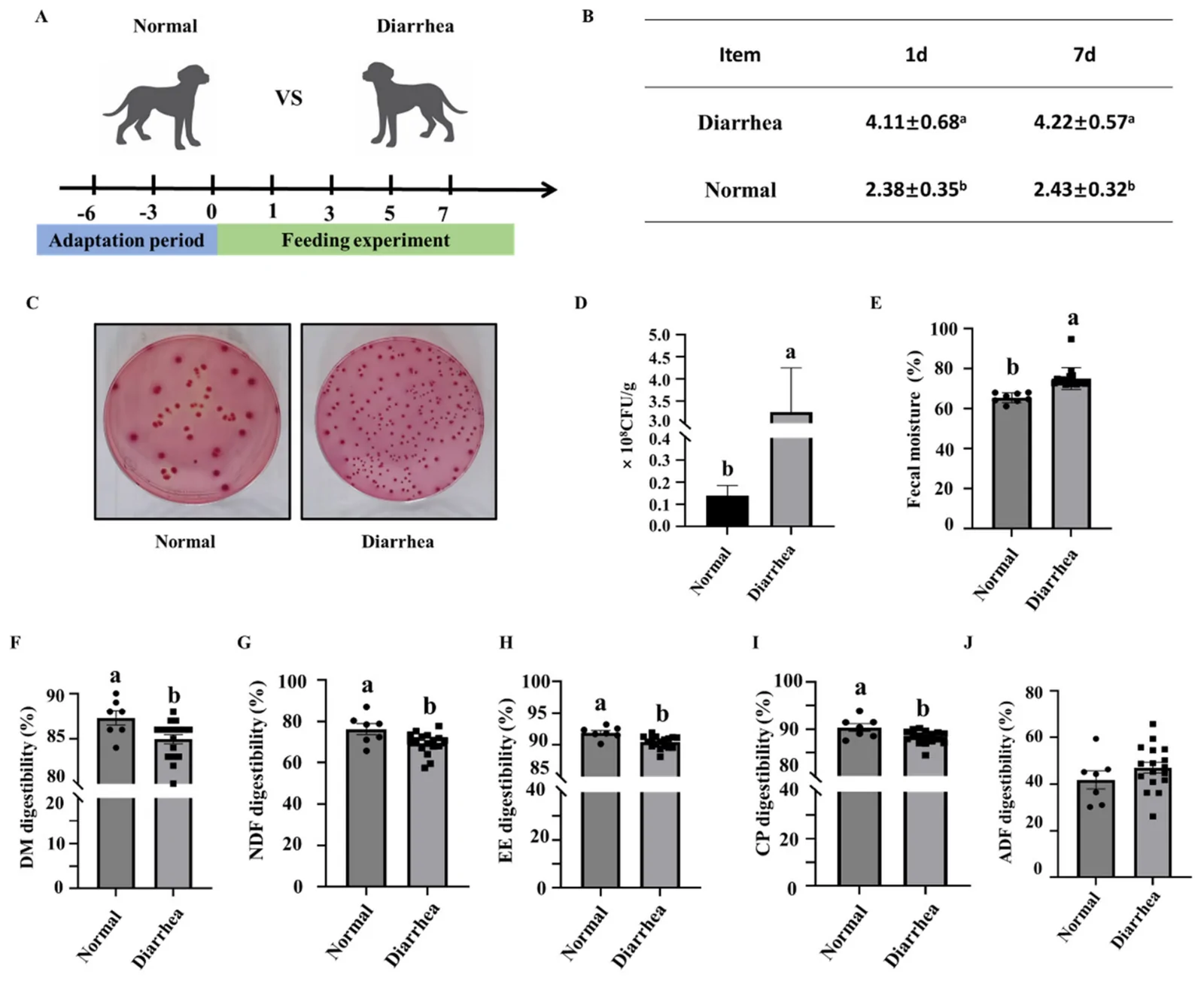
Differences in fecal scores, the number of pathogenic bacteria in the feces, and the digestibility of nutrients between healthy dogs and dogs with intestinal sensitivity. (**A**) Schematic diagram of the trial; (**B**) Fecal score; (**C**,**D**) The number of pathogenic bacteria in feces; (**E**) Fecal moisture; (**F**) DM digestibility; (**G**) NDF digestibility; (**H**) EE digestibility; (**I**) CP digestibility; (**J**) ADF digestibility. Data are presented as the mean ± SEMs (n ≥ 7). Different letters indicate significant differences (*p* < 0.05).

The Waltham Feces Scoring System (WFS) was used to evaluate the feces of each dog in the control or dogs affected with intestinal sensitivity. As shown, the fecal scores of dogs affected with intestinal sensitivity were higher than those of the control dogs during the experimental period (Figure 1B). MacConkey (MCA) agar was employed to enumerate the microorganisms in fecal samples, and we found that the number of pathogenic microorganisms in the intestinal-sensitive dogs was higher than that of control dogs (Figure 1C,D). Fecal moisture content in healthy dogs was lower than that in intestinal sensitive dogs (Figure 1E). Further study demonstrated that the digestibility of dry matter (DM), neutral detergent fiber (NDF), crude fat (EE), and crude protein (CP) was higher in healthy dogs than in the intestinal-sensitive dogs (Figure 1F–J).

### 3.2. The Ameliorative Effect of Fecal Microbiota Transplantation on Fecal Score and Nutrient Digestibility in Dogs with Intestinal Sensitivity

**Figure 2:**
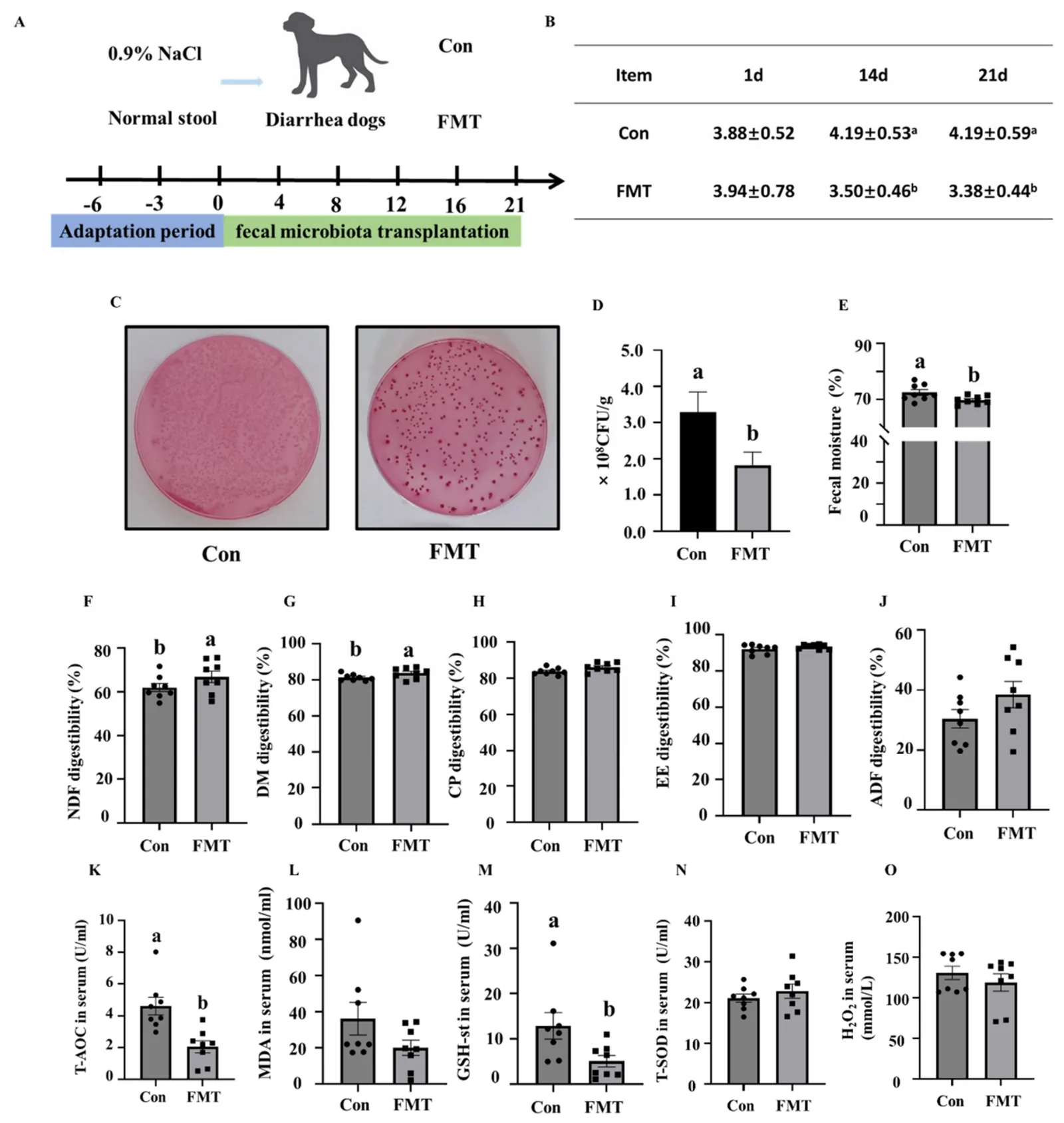
The ameliorative effect of fecal microbiota transplantation from healthy dogs on dogs with intestinal sensitivity. (**A**) Schematic diagram of the trial; (**B**) Fecal score; (**C**,**D**) The number of pathogenic bacteria in feces; (**E**) Fecal moisture; (**F**) NDF digestibility; (**G**) DM digestibility; (**H**) CP digestibility; (**I**) EE digestibility; (**J**) ADF digestibility. (**K**) T-AOC in serum; (**L**) MDA in serum; (**M**) GSH-st in serum; (**N**) T-SOD in serum; (**O**) H_2_O_2_ in serum. Data are presented as the mean ± SEM (n = 8). Different letters indicate significant differences (*p* < 0.05).

As expected, FMT treatment resulted in improved fecal scores of dogs with diarrhea at both 14d and 21d post-FMT (Figure 2B). Also, the number of pathogenic bacteria in the feces of the dogs that received microorganisms from the intestines of healthy dogs was significantly decreased at the end of the trial (Figure 2C,D). Apparent nutrient digestibility was determined and we found that the digestibility of DM and NDF was significantly enhanced following FMT treatment as compared with that of the controls. Fecal moisture content was significantly reduced in the FMT group compared with the Con group (Figure 2E–J), and the serum levels of glutathione S-transferase (GSH-ST) and total antioxidant capacity (T-AOC) exhibited a significant decline in the dogs receiving FMT. In contrast, the contents of MDA, T-SOD and H_2_O_2_ in the serum did not show significant changes before and after the treatment (Figure 2K–O).

### 3.3. The Effect of Fecal Microbiota Transplantation on Serum Biochemical Parameters and Complete Blood Count

**Figure 3:**
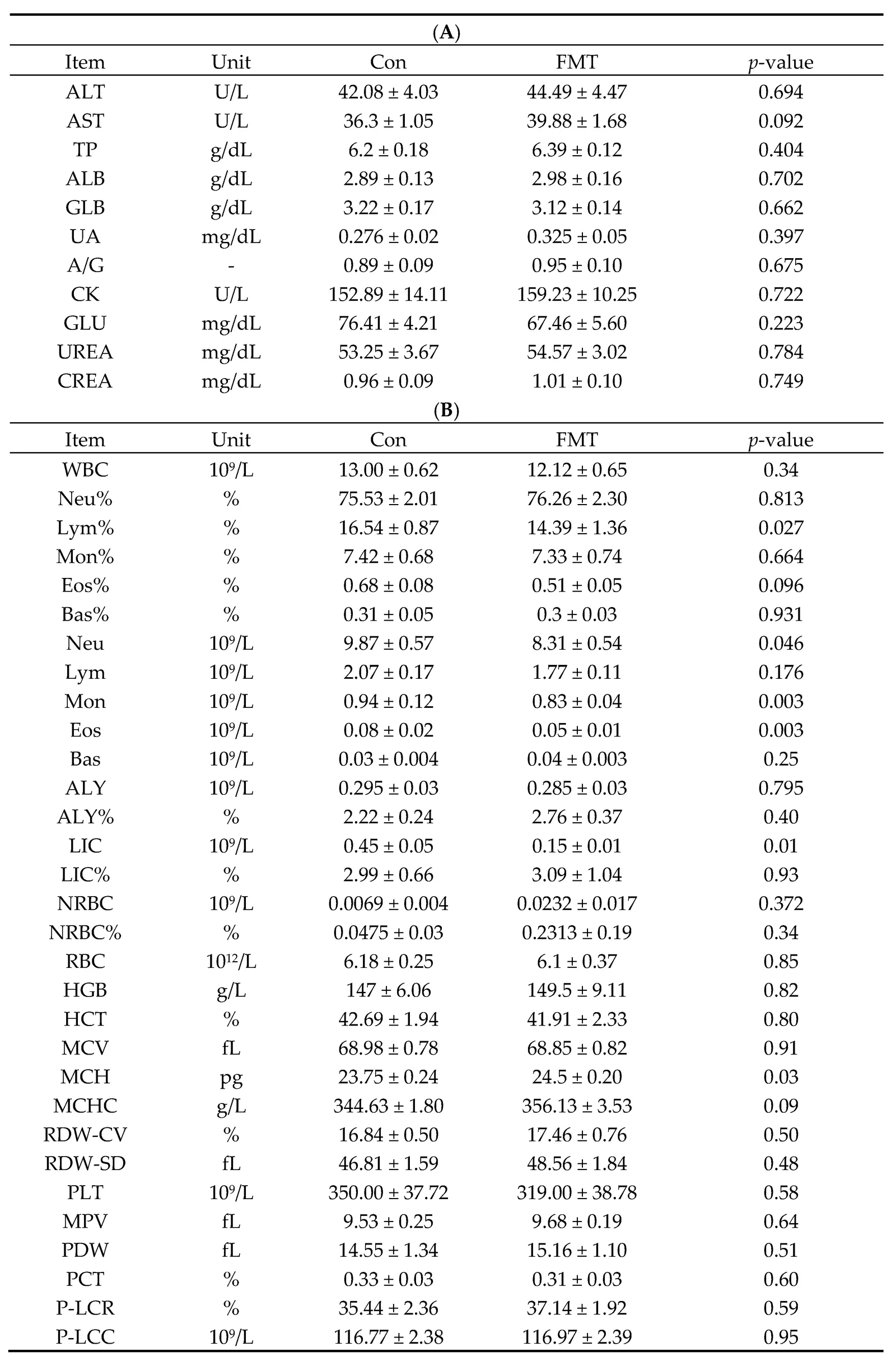
The effect of fecal microbiota transplantation on serum biochemical parameters and complete blood count. (**A**) Serum biochemical indicators; (**B**) Complete Blood Count. Data are presented as the mean ± SEM (n = 8).

The serum biochemical parameters remained largely unaffected, with no significant alterations observed between groups (Figure 3A). Notably, the FMT group exhibited a markedly lower lymphocyte percentage (Lym%) compared with the Con group, accompanied by significant reductions in the absolute counts of neutrophils (Neu), monocytes (Mon), eosinophils (Eos), and large immature cells (LIC). Among the red blood cell-related indicators, the mean corpuscular hemoglobin content (MCH) in the FMT group significantly increased. The other routine blood indicators such as total white blood cell count (WBC), total red blood cell count (RBC), hemoglobin (HGB), hematocrit (HCT), and platelet count (PLT) showed no significant differences between the two groups (Figure 3B). Collectively, these findings indicated that certain dominant microorganisms harbored in healthy dogs are capable of ameliorating the physiological status of dogs with intestinal sensitivity.

### 3.4. The Differences Between the Intestinal Microbiota of Healthy Dogs and That of Dogs with Intestinal Sensitivity

**Figure 4:**
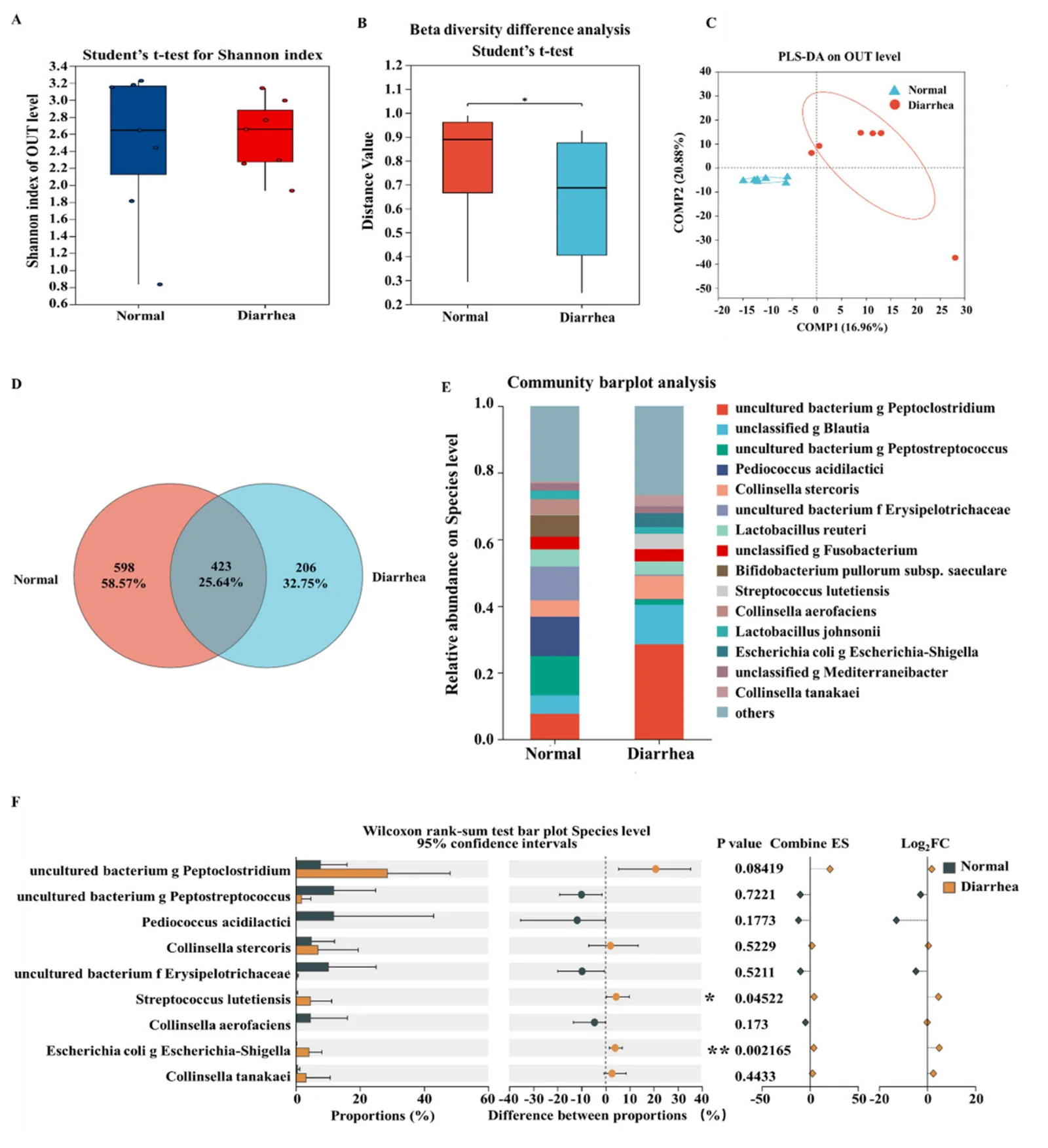
The differences between the intestinal microbiota of healthy dogs and that of dogs with intestinal sensitivity. (**A**) Shannon index; (**B**) Beta diversity difference analysis between normal dogs and those with diarrhea; (**C**) PLS-DA; (**D**) Venn diagram between normal dogs and those with diarrhea. (**E**) Relative abundance at the species level; (**F**) Wilcoxon rank-sum test bar plot at the species level. Data are presented as the mean ± SEM (n = 6). * *p* < 0.05, ** *p* < 0.01.

Subsequently, we compared the gut microbial composition between healthy dogs and dogs with intestinal sensitivity. Notably, the Shannon index—a metric of alpha diversity—was significantly higher in the healthy group than in the intestinal-sensitive group (Figure 4A). Beta-diversity metrics revealed separation between groups, with the Diarrhea group displaying significantly lower beta diversity relative to the Normal group (Figure 4B). Partial Least Squares Discriminant Analysis (PLS-DA) demonstrated that fecal microbial samples from healthy dogs formed tight clusters, whereas samples from dogs with intestinal sensitivity exhibited greater scattering (Figure 4C). The Venn diagram illustrated 598 unique OTUs detected in the Normal group versus 206 unique OTUs in the Diarrhea group (Figure 4D). Compared with healthy dogs, the intestinal-sensitive group had elevated relative abundances of *Peptoclostridium*, *Blautia*, and *Escherichia-Shigella*, alongside reduced levels of beneficial taxa such as *Pediococcus acidilactici* and *Peptostreptococcus* (Figure 4E). At the species level, *Pediococcus acidilactici* was more abundant in the Normal group, while *Shigella* was significantly enriched in the Diarrhea group (Figure 4F).

### 3.5. Pediococcus acidilactici Demonstrates a Beneficial Effect in Alleviating Intestinal Sensitivity in Dogs

**Figure 5:**
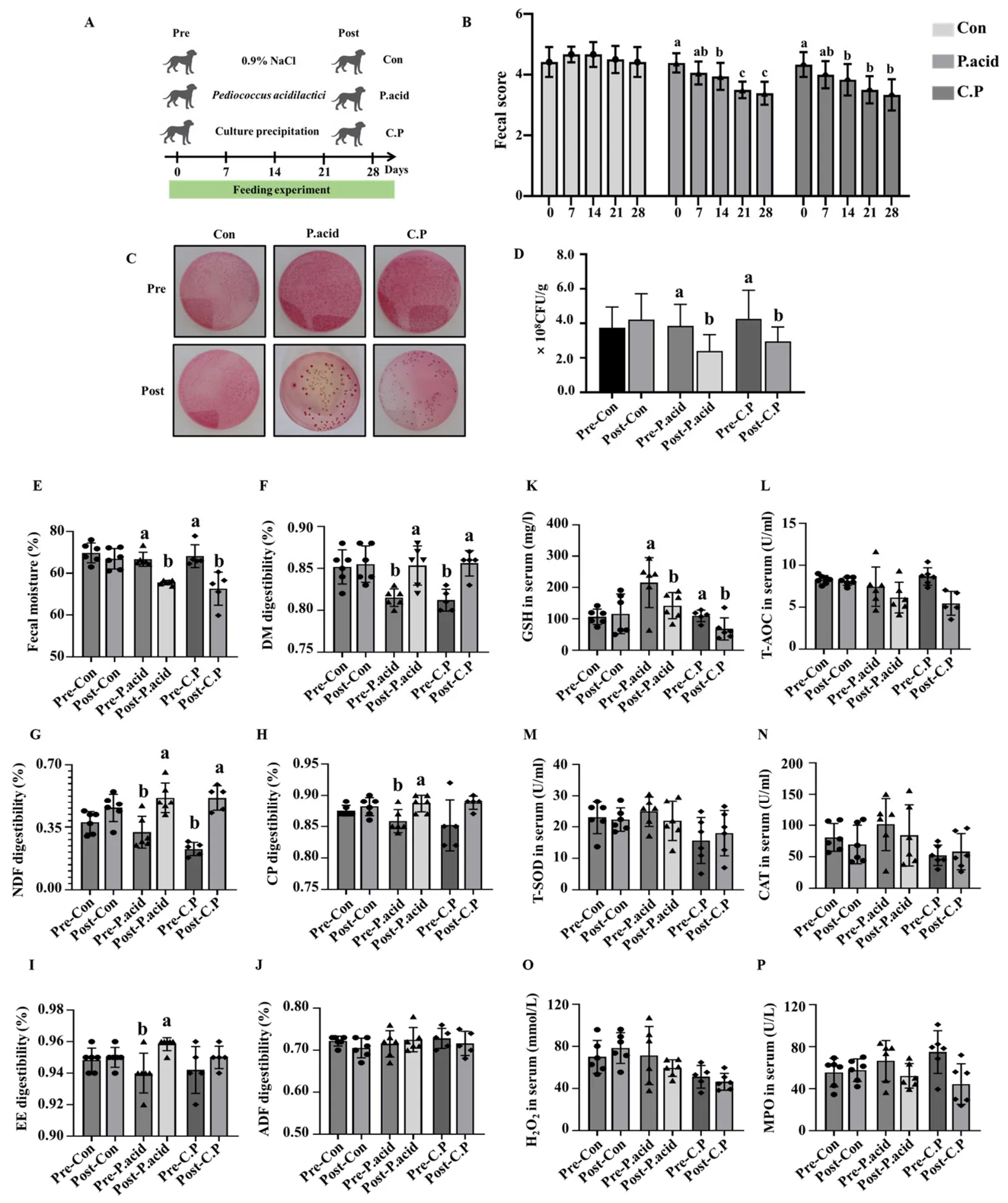
*Pediococcus acidilactici demonstrates a beneficial effect in alleviating diarrhea in dogs*. (**A**) Schematic diagram of the trial; (**B**) Fecal score; (**C**,**D**) The number of pathogenic bacteria in feces; (**E**) Fecal moisture; (**F**) DM digestibility; (**G**) NDF digestibility; (**H**) CP digestibility; (**I**) EE digestibility; (**J**) ADF digestibility; (**K**) GSH in serum; (**L**) T-AOC in serum; (**M**) T-SOD in serum; (**N**) CAT in serum; (**O**) H_2_O_2_ in serum; (**P**) MPO in serum. Data are presented as the mean ± SEM (n = 6). Different letters indicate significant differences (*p* < 0.05).

To explore a beneficial effect of *P. acidilactici* Long21 supplementation on intestinal health, dogs affected by intestinal sensitivity were supplemented with saline or *P. acidilactici* Long21 for 28 days. The fecal scores of intestinal sensitive dogs were compared before and after supplementation. Throughout the experimental period, fecal scores in the Con group showed no improvement at all. In contrast, both the *P. acid* and CP groups demonstrated significantly reduced fecal scores on days 14, 21, and 28 compared to the baseline (Figure 5B). In the Con group, there was no significant difference in the composition of the microbial community in the dogs’ feces before and after being fed with normal saline. However, in the *P. acid* group and C. P group, the number of microorganisms in the dogs’ feces was significantly lower at the end of the experiment compared to the initial level (Figure 5C,D). Throughout the experimental period, the Con group exhibited no significant alterations in fecal moisture content or in the apparent digestibility of dry matter (DM), neutral detergent fiber (NDF), ether extract (EE), and crude protein (CP). By contrast, the *P. acid* and CP groups demonstrated a marked decrease in fecal moisture content, along with concurrent improvements in the apparent digestibility of DM, NDF and EE to varying extents (Figure 5E–J). Compared with the pre-experiment levels, the serum antioxidant indices (GSH, T-AOC, T-SOD, CAT, H_2_O_2_, MPO) in the Con group showed no significant changes after the experiment. By contrast, the *P. acid* group exhibited a significant decrease in GSH, while the other indices showed a general downward trend, albeit without statistical significance (Figure 5K–P).

### 3.6. Effect of P. acidilactici Long21 Supplementation on Serum Biochemical Parameters and Complete Blood in Intestinal Sensitive Dogs

**Figure 6:**
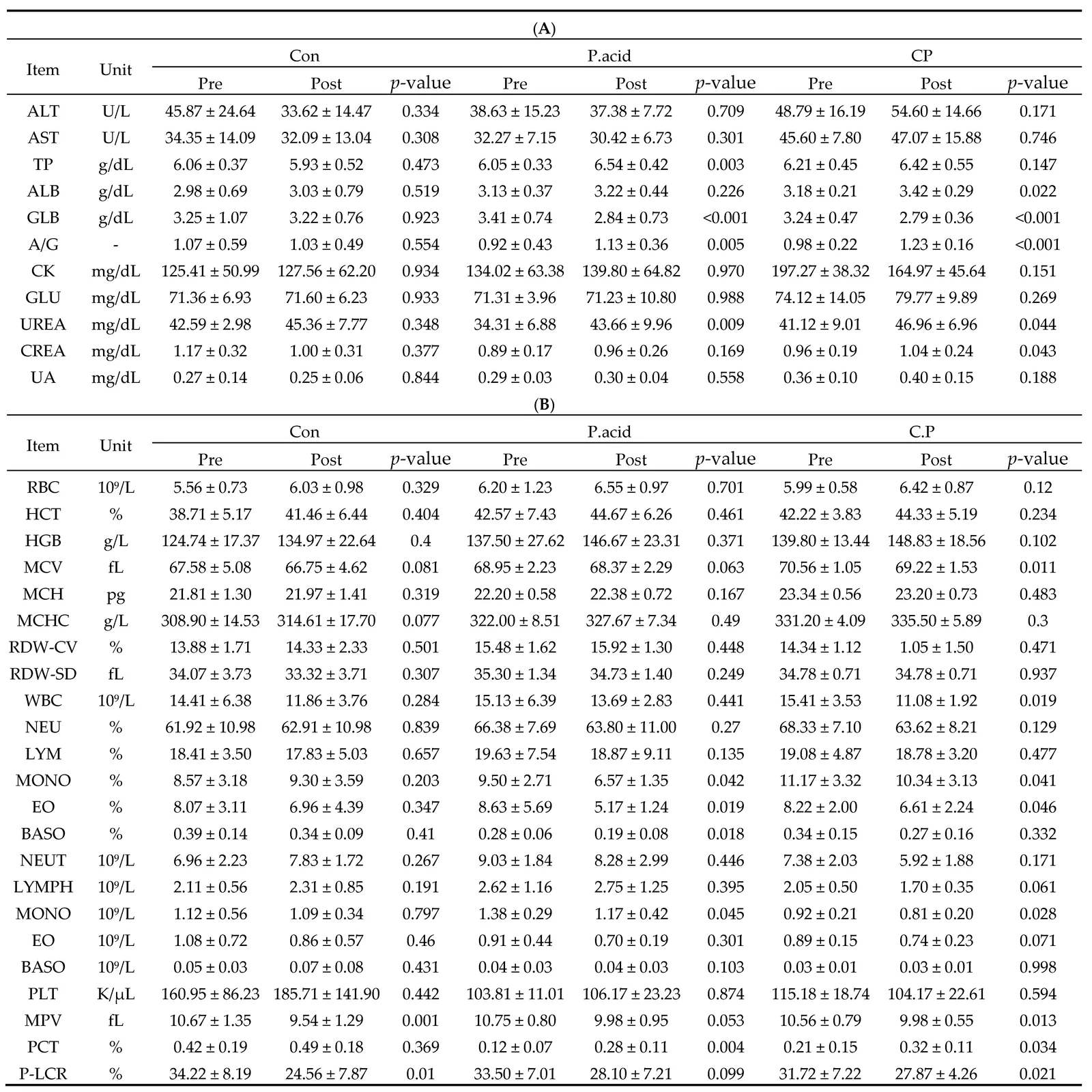
*Pediococcus acidilactici* demonstrates a beneficial effect in alleviating intestinal sensitivity in dogs on serum biochemical parameters and complete blood count. (**A**) Serum biochemical indicators; (**B**) Complete Blood Count. Data are presented as the mean ± SEM (n = 6).

In the Con group, all measured serum biochemical indices remained stable, with no significant changes observed between the Pre- and Post-treatment periods. In the P. acid group, however, post-treatment levels of TP and UREA were significantly higher than pre-treatment values, while GLB was significantly lower. These alterations collectively led to a significant increase in the A/G ratio at Post-treatment. No significant differences were found in other parameters (ALT, AST, ALB, CK, GLU, CREA, UA) between pre- and post-treatment. In the CP group, significant increases in ALB, A/G ratio, UREA and CREA were observed after probiotic administration, whereas GLB was significantly reduced. In contrast, the remaining indices (ALT, AST, TP, CK, GLU, UA) were not affected by probiotic supplementation (Figure 6A).

The complete blood count indicators of the Con group remained stable before and after the intervention, with only MPV and P-LCR showing significant decreases. After the intervention in the P. acid group, MCV showed a downward trend, while MONO%, EO%, BASO%, and MONO significantly decreased, accompanied by a significant increase in PCT. In the CP group after the intervention, MCV, WBC, MONO%, EO%, MONO, and P-LCR significantly decreased, whereas MPV and PCT were significantly elevated after the intervention (Figure 6B).

### 3.7. Untargeted Metabolomics Analysis of Dog Feces After Feeding with Pediococcus acidilactici

**Figure 7:**
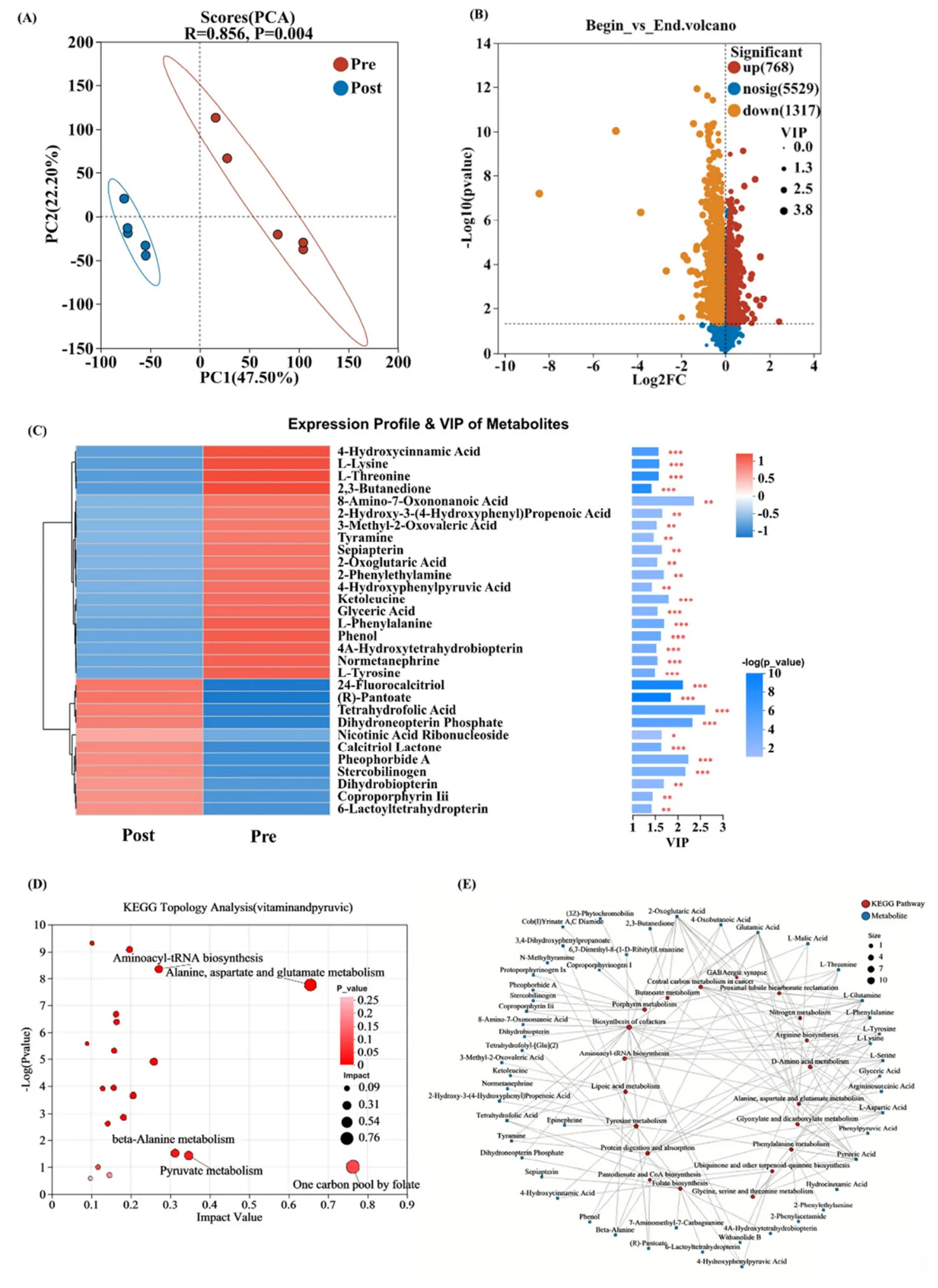
Non-targeted metabolomics analysis of dogs with intestinal sensitivity after feeding with Pediococcus acidilactici. (**A**) PCA; (**B**) Volcanic maps; (**C**) Expression profile & VIP of metabolites; (**D**) KEGG Topology analysis; (**E**) KEGG pathway enrichment analysis network diagram; Data are presented as the mean ± SEM (n = 5). * *p* < 0.05, ** *p* < 0.01, *** *p* < 0.001.

Principal Component Analysis (PCA) revealed complete separation between pre- and post-treatment groups along the PC1 dimension (accounting for 47.5% of variance), with model fit R^2^ = 0.856 and statistical significance *p =* 0.004 (Figure 7A). Collectively, these findings robustly confirmed that the metabolic profile differed before and after *P. acidilactici* intervention, supporting the consistency of the intervention effect. Subsequent Volcano plot analysis identified 2085 significantly altered metabolites (fold change > 2, VIP > 1, *p* < 0.05) in both positive and negative ion modes, comprising 1317 down-regulated and 768 up-regulated metabolites (Figure 7B). VIP analysis further refined this to 28 differential metabolites, from which 20 key metabolites were selected, including nicotinic acid ribonucleoside, glutamine, serine, sepiapterin and tyramine (Figure 7C). These key metabolites are functionally enriched in critical pathways such as pyruvate metabolism, one-carbon pool by folate metabolism, alanine-aspartate-glutamate metabolism, aminoacyl-tRNA biosynthesis, and beta-alanine metabolism (Figure 7D). Network map analysis demonstrated that differential metabolites were predominantly enriched in several core metabolic pathways, namely amino acid metabolism, energy metabolism, cofactor biosynthesis, and porphyrin metabolism, indicating that *Pediococcus acidilactici* intervention significantly disrupted these critical pathways. Within the metabolic network, glutamate, malate, and pyruvate emerged as central hub nodes, underscoring their potential as key regulators of systemic metabolic balance. As such, these core metabolites warrant prioritization in subsequent mechanistic research and the design of targeted intervention strategies (Figure 7E).

### 3.8. Correlation Analysis Between Metabolites and Nutrient Digestibility

**Figure 8:**
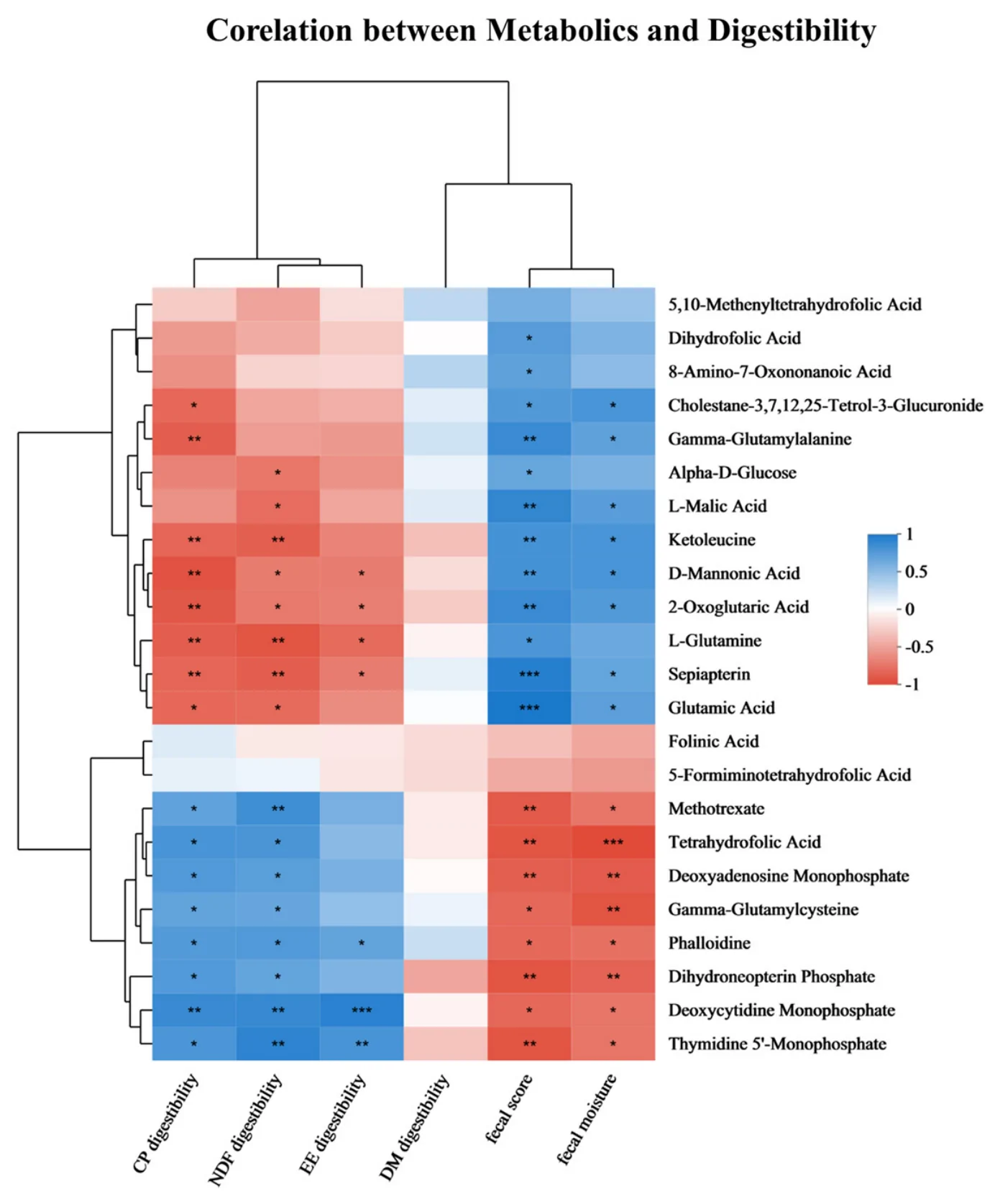
Correlation between metabolites and digestibility. * *p* < 0.05, ** *p* < 0.01, *** *p* < 0.001.

To further elucidate the metabolic regulatory mechanisms underlying nutrient utilization, correlations between differential metabolites and key digestive physiological indicators were assessed and correlations between metabolites and four core nutrient digestibility rates (CP, NDF, EE, DM), as well as fecal scores and fecal moisture, were observed (Figure 8). The results revealed that distinct metabolic clusters were significantly associated with specific digestive traits. Metabolites such as glutamic acid, L-malic acid, and 2-oxoglutaric acid exhibited strong positive correlations with CP digestibility, while showing significant negative correlations with fecal scores and fecal moisture. Conversely, metabolites including deoxycytidine monophosphate, thymidine 5′-monophosphate, and tetrahydrofolic acid were significantly negatively correlated with CP, NDF, and DM digestibility, but positively correlated with fecal indicators. Collectively, these findings underscore the pivotal role of core metabolites in regulating nutrient digestion efficiency, providing valuable evidence for the development of targeted intervention strategies.

## 4. Discussion

Intestinal health is tightly linked to the structure and function of the gut microbiota, and disturbances in microbial homeostasis are widely recognized as key drivers of intestinal dysfunction in companion animals [26,27]. In dogs, intestinal sensitivity frequently presents as intermittent soft feces, diminished nutrient digestibility, and low-grade intestinal inflammation. Nevertheless, the specific microbial signatures and associated metabolic mechanisms underlying these manifestations remain largely elusive [28,29]. In this study, dogs with naturally occurring intestinal sensitivity exhibited significantly higher fecal scores, elevated fecal pathogenic bacterial counts, and markedly reduced apparent digestibility of DM, CP, NDF and EE compared with healthy dogs, accompanied by increased fecal moisture. Such phenotypic alterations were closely associated with impaired intestinal barrier function, reduced digestive enzyme activity, and disrupted nutrient absorption, which are characteristic features of gut dysbiosis-related disorders [30,31]. The overgrowth of opportunistic pathogens was associated with enhanced intestinal inflammatory responses and epithelial damage, and it appeared to aggravate diarrhea effects and reduce feed utilization efficiency [32,33]. Collectively, these findings suggest that canine intestinal sensitivity is closely associated with a dual interplay of physiological dysfunction and microbial dysbiosis.

To verify the causal role of the gut microbiota in the development of intestinal sensitivity, fecal microbiota transplantation from healthy dogs was performed. After FMT administration, dogs with intestinal sensitivity exhibited significantly improved fecal consistency, reduced pathogenic bacterial load, enhanced nutrient digestibility, and decreased fecal moisture. Additionally, FMT mitigated systemic oxidative stress and inflammatory responses, as reflected by reduced serum GSH-ST and T-AOC levels and diminished inflammatory cell populations, including lymphocytes, eosinophils, and neutrophils. These findings are consistent with previous evidence indicating that FMT is associated with restored microbial homeostasis, inhibited pathogenic colonization, attenuated intestinal inflammation, and enhanced digestive function [34,35,36]. Notably, serum AOC and GSH were decreased in FMT-treated dogs. The diarrheic recipient dogs exhibited sustained intestinal inflammation and oxidative stress before receiving the intervention, which was associated with a compensatory increase in systemic antioxidant defence. After FMT treatment, the remodelled gut microbiota was associated with relieved intestinal inflammation and reduced gut-originated oxidative stress, which in turn appeared to lower serum AOC and GSH towards the physiological range of healthy dogs. In summary, these findings indicate an association between the gut microbiota and intestinal sensitivity and suggest that reconstructing a healthy microbial community may represent a viable strategy for reversing associated physiological dysfunction.

Additionally, 16S rRNA gene sequencing further revealed striking disparities in gut microbial composition between healthy dogs and those with intestinal sensitivity. Notably, dogs with diarrhea exhibited significantly lower alpha diversity, represented by the Shannon index, and clearly distinguishable beta diversity patterns when compared with their healthy counterparts. PLS-DA analysis showed clear separation between the two groups, indicating a highly divergent microbial community structure. In addition, the diarrhea group harboured significantly fewer unique OTUs than the healthy group, suggesting compromised microbial richness and a corresponding loss of functional redundancy. A depleted microbial diversity often weakens colonization resistance against pathogens and reduces the ability of the gut ecosystem to maintain stability, which may increase susceptibility to intestinal sensitivity [37,38,39].

At the species level, the relative abundances of *Peptoclostridium*, *Blautia*, and *Escherichia-Shigella* were significantly increased in dogs with intestinal sensitivity, whereas beneficial bacteria, particularly *P. acidilactici*, were reduced. *Escherichia-Shigella* is a well-documented opportunistic pathogen that can disrupt intestinal tight junctions, trigger mucosal inflammation, and induce diarrhea [40]. *Peptoclostridium* and *Blautia* have been associated with proteolytic fermentation and inflammatory bowel conditions in humans and animals, and their enrichment may contribute to gut barrier dysfunction [41,42]. In contrast, *P. acidilactici*, a well-studied lactic acid bacterium, possesses multiple probiotic properties, including the ability to inhibit pathogenic microorganisms, modulate host immune responses, and strengthen intestinal barrier integrity [43]. Notably, its substantial depletion in dogs with intestinal sensitivity suggests that the loss of this key beneficial microbe may be a contributing factor to gut dysfunction. These findings define a distinct microbial signature associated with canine intestinal sensitivity and position *P. acidilactici* as a potential keystone species worthy of further investigation.

Given the results of the comparative microbiota analysis, we further investigated the probiotic effects of *P. acidilactici* in dogs with intestinal sensitivity. Supplementation with live *P. acidilactici*, either alone or in combination with fermentation supernatant, markedly improved fecal scores, reduced fecal moisture, decreased pathogenic bacterial counts, and enhanced the digestibility of DM, NDF, and EE. Moreover, *P. acidilactici* supplementation exerted notable immunomodulatory effects on systemic immune homeostasis, as indicated by lowered serum globulin levels, an improved albumin/globulin ratio, and a reduction in circulating monocyte counts. These changes were associated with relieved systemic inflammation and restored immune balance. The similar improvements observed in dogs receiving live bacteria with or without the fermentation supernatant suggest that the viable bacterial cells play a central role in the probiotic action. These findings suggest that *P. acidilactici* supplementation is associated with improvements in canine intestinal sensitivity, potentially through a synergistic interplay between antimicrobial activity, enhanced digestive efficiency and immunomodulatory regulation.

To explore the underlying metabolic mechanisms, untargeted fecal metabolomics was conducted. PCA results showed distinct separation between pre- and post-intervention groups, indicating that P. acidilactici supplementation was associated with substantial remodelling of the fecal metabolic landscape. A total of 2085 differentially abundant metabolites were identified, with key metabolites, including nicotinic acid ribonucleoside, glutamine, serine, sepiapterin, and tyramine, significantly altered. Functional enrichment analysis revealed that these metabolites were mainly involved in pyruvate metabolism, the one-carbon pool by the folate pathway, alanine–aspartate–glutamate metabolism, aminoacyl-tRNA biosynthesis, and beta-alanine metabolism. Among them, glutamine acts as a preferential energy source for intestinal epithelial cells and promotes mucosal healing, and amino acid metabolism is essential for sustaining protein synthesis, optimizing nutrient utilization, and modulating immune responses [44,45]. Metabolic network analysis further identified glutamate, malate, and pyruvate as hub metabolites involved in central energy and nitrogen metabolism [46,47,48]. These results suggest that *P. acidilactici* supplementation is associated with improved intestinal health, possibly through multifaceted changes encompassing the gut microbial architecture and host metabolic pathways related to energy supply, mucosal repair, and nutrient utilization.

Correlation analysis between differential metabolites and nutrient digestibility provided further insights into the mechanism by which *P. acidilactici* improves digestive efficiency. Metabolites including glutamic acid, L-malic acid, and 2-oxoglutaric acid were strongly positively correlated with crude protein digestibility and negatively correlated with fecal scores and moisture. In contrast, deoxycytidine monophosphate, thymidine 5′-monophosphate, and tetrahydrofolic acid showed negative correlations with nutrient digestibility and positive correlations with diarrheic phenotypes [49,50,51]. These associations suggest that key metabolites may serve as signaling molecules linking the gut microbiota and host digestive physiology. Correlated changes in these metabolic pathways appeared to accompany enhanced nutrient absorption, suppressed intestinal inflammation, and improved fecal quality, although causal relationships remain to be confirmed.

Notwithstanding the strengths of this study, several limitations merit careful consideration. First, the relatively small sample size represents a common practical constraint for intervention trials using client-owned companion dogs; this modest sample size may have restricted statistical power for detecting subtle yet meaningful alterations. Although post hoc calculation revealed moderate-to-large effect sizes for most key readouts, no a priori power analysis was performed, and larger-sample-size multi-centre trials are required to further validate the present findings and improve generalizability. Second, our analysis was limited to fecal microbiota and metabolites, leaving mucosal-associated microbial communities and intestinal transcriptomic profiles uninvestigated. Additionally, the exact molecular mechanisms through which *P. acidilactici* orchestrates metabolic pathway regulation and epithelial function remain incompletely understood. Future investigations leveraging expanded sample sizes, extended intervention periods, and multi-omics integrative approaches will be critical to comprehensively unravel the tripartite interactions between *P. acidilactici*, the gut microbiome, and host metabolism.

## 5. Conclusions

In conclusion, canine intestinal sensitivity is characterized by gut microbial dysbiosis, reduced microbial diversity, pathogenic bacteria overgrowth, impaired nutrient digestibility, and disturbed metabolic homeostasis. *P. acidilactici* Long21 effectively alleviates intestinal dysfunction by rebalancing the gut microbiota, suppressing pathogenic colonization, orchestrating core metabolic pathways, and improving nutrient utilization. The beneficial effects observed, as well as the metabolomics data, supported the regulatory role of *P. acidilactici* in intestinal health and metabolism in dogs affected by intestinal sensitivity, thereby providing a theoretical basis for the application of *P. acidilactici* as a promising probiotic strategy for maintaining gut health in dogs.

## Data Availability

All data needed to assess the conclusions in this study are provided in the manuscript and/or the Supplementary Materials. The data used in this study are available from the Science Data Bank and can be accessed via https://doi.org/10.57760/sciencedb.39238 and https://doi.org/10.57760/sciencedb.39236.

## Supplementary Materials

The following supporting information can be downloaded at: https://www.mdpi.com/article/10.3390/ani16203181/s1, Table S1: The detailed ingredient list and nutritional composition of the commercial chicken-brown rice complete dog diet (Fupaise Co., Ltd.).; Table S2: Summary of differentially expressed metabolites identified from canine fecal metabolomics.

## Abbreviations

ADF	Acid Detergent Fiber
CAT	Catalase
CP	Crude Protein
DM	Dry Matter
EE	Ether Extract
GSH-ST	Glutathione S-transferase
H_2_O_2_	Hydrogen peroxide
MDA	Malondialdehyde
MPO	Myeloperoxidase
NDF	Neutral Detergent Fiber
