# Supplementary material for: Pediococcus acidilactici Long21 Alleviates Intestinal Sensitivity and Improves Gut Health in German Shepherd Dogs by Regulating Intestinal Metabolic Profiles
Source: Animals (Basel). 2026 Oct 10;16(20):3181. doi: 10.3390/ani16203181 (PMC13700620; doi:10.3390/ani16203181)
Supplement: Supplementary file 1 [file animals-16-03181-s001.zip › animals-4576950-supplementary Table S1.pdf]

Supplementary Table S1. The detailed ingredient list and nutritional composition of the commercial chicken-brown rice complete dog diet (Fupaise Co., Ltd.).

Ingredients	Content (%)	Composition	%
Fresh chicken	32	Moisture	⩽10.0%
Frozen chicken	20	Crude protein	⩾28.0%
Brown rice	18	Crude fat	⩾14.0%
Chicken oil	8	Crude fiber	⩽8.0%
Deep-sea fish oil	6	Coarseash	⩽10.0%
Fish meal	3	Calcium	⩾1.0%
Green peas	3	Phosphoru	⩾0.8%
Chicken liver powder	2	Water soluble chloride	⩾0.09%
other ingredient	8	L-lysine	⩾1.0%
